# Supplementary material for: Efficient differential expression analysis of large-scale single-cell transcriptomics data using Dreamlet
Source: Nat Commun. 2026 Sep 23;17:9597. doi: 10.1038/s41467-026-75680-8 (PMC13601538; doi:10.1038/s41467-026-75680-8)
Supplement: Supplementary file 2 [file 41467_2026_75680_MOESM2_ESM.pdf]

nature portfolio

Peer Review File

Efficient differential expression analysis of large-scale single cell transcriptomics data using dreamlet

Corresponding Author: Dr Gabriel Hoffman

This manuscript has been previously reviewed at another journal. This document only contains information relating to versions considered at Nature Communications.

This file contains all reviewer reports in order by version, followed by all author rebuttals in order by version.

Version 1:

Reviewer comments:

Reviewer #1

(Remarks to the Author)
I have carefully reviewed the revised manuscript and the authors' detailed responses to the reviewers. Overall, the authors have made substantial efforts to address the comments, and I find the revisions constructive and largely satisfactory.

Regarding my first comment on benchmarking against a naive baseline, I commend the authors for their efforts in providing an additional comparison. They implemented a straightforward pseudobulk routine and reported runtime and memory usage, which aligns well with the spirit of my original suggestion. This addition is helpful in demonstrating performance advantages of their method over a simple alternative. I note, however, that while the runtime benefits of dreamlet over the naive approach are clear, the memory usage appears quite similar between the two in the presented results. It may be useful for the authors to make this point explicit in the text.

On my second comment regarding batch effect handling, I commend the authors for extending their analysis by incorporating Harmony that I previously suggested as a covariate and carefully considering 10x pool effects. The added analysis directly addresses my concern by demonstrating that batch-related variation can be effectively mitigated and by showing that modeling technical sources of variation improves the interpretability of the biological signal.

I also reviewed the authors' responses to the remaining comments (i.e., from reviewer #3). These commments were relatively minor, namely the request to extend the range of simulated cell counts and to expand benchmarking using a second dataset. Both points have been addressed in the revision: the simulations now include as few as 20 cells per donor, and the benchmarking has been extended to compare dreamlet, limma, and DESeq2 across both the primary Alzheimer's dataset and the Mathys et al. dataset. These additions are appropriate and strengthen the manuscript by highlighting performance under sparse conditions and demonstrating that dreamlet achieves the highest or equal-highest replication across datasets. The missing legend in Supplemental Figure 2 has also been corrected.

(Remarks on code availability)
I previously reviewed the GitHub code and did not repeat this evaluation in the current round.

Open Access This Peer Review File is licensed under a Creative Commons Attribution 4.0 International License, which permits use, sharing, adaptation, distribution and reproduction in any medium or format, as long as you give appropriate credit to the original author(s) and the source, provide a link to the Creative Commons license, and indicate if changes were made.
In cases where reviewers are anonymous, credit should be given to 'Anonymous Referee' and the source.
The images or other third party material in this Peer Review File are included in the article's Creative Commons license, unless indicated otherwise in a credit line to the material. If material is not included in the article's Creative Commons license and your intended use is not permitted by statutory regulation or exceeds the permitted use, you will need to obtain permission directly from the copyright holder.
To view a copy of this license, visit https://creativecommons.org/licenses/by/4.0/
